# Literary Notes

**Published:** 1903-11

**Authors:** 


### LITERARY NOTES.

The Pathology of the Typhoid Ulcer is the title of a booklet just issued by The Arlington Chemical Co., of Yorkers, N. Y. It contains four well executed plates, printed in colors showing: (1) swollen Peyer's patches; (2) superficial necrosis; (3) deep ulceration; (4) cicatrisation. The descriptive text, comprising some seven or eight pages, is of scientific interest and accuracy. The illustrations could not be improved upon. The brochure will be sent to any physician upon application to the publishers at above address.

---

A Diagnostic Chart of Tumors and Pseudotumors.—Battle & Co. have just issued a very useful chart of tumors, true and false, compiled by Dr. Edward C. Hill from standard works on surgery and pathology. The subject matter is divided into solid neoplasma (subdivided into benign and malignant growths) and true and false cysts. The general characteristics of each division are given, and their 24 classes, embracing over 100 varieties, are compared critically in columns under the following headings: tissue, topography, number, size, conformation, color, consistence, mobility, sensibility, surrounding tissues, occurrence, history of growth, and miscellaneous points. Features of special differential value are emphasised by the use of italics. The object of this chart is to show at a glance for ready comparison much that could be learned in a diagnostic way only from the perusal of hundreds of pages of ordinary text. It stands to such books as an atlas does to a gazetteer. This very convenient and valuable compendium is at the command gratis of any and every practitioner of medicine, who will take the trouble of writing a postal card to Battle & Co., 2001 Locust street, Saint Louis.

